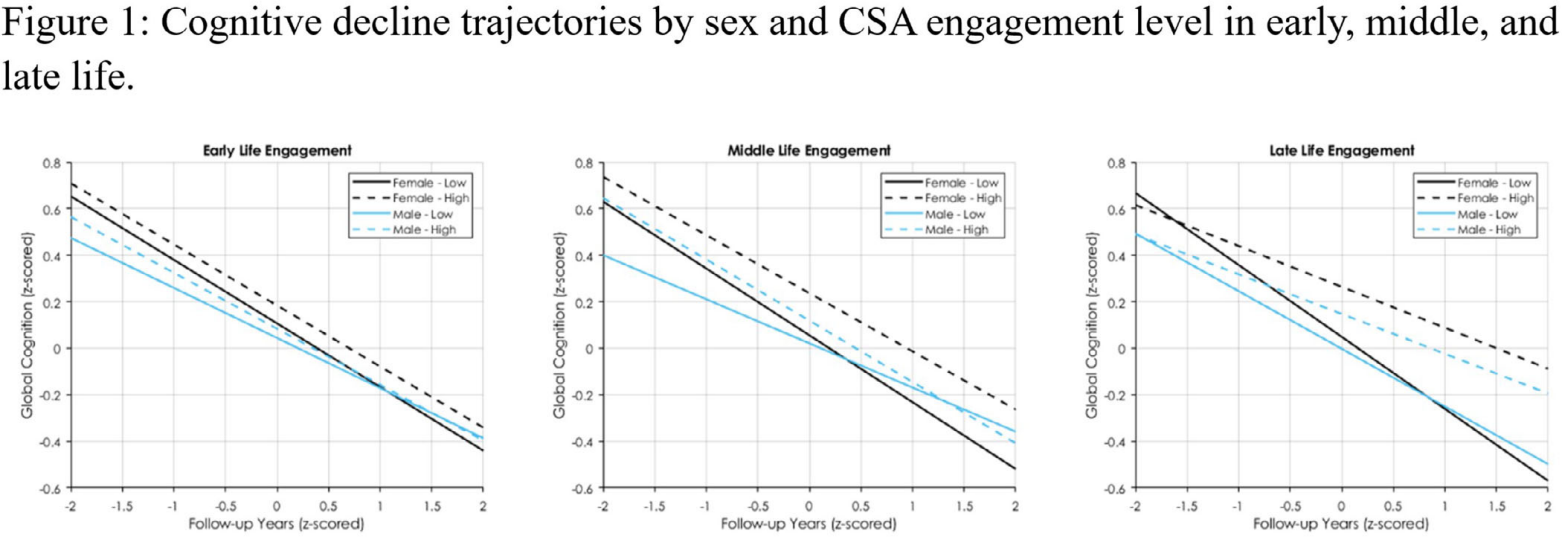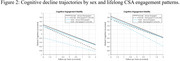# The Impact of Cognitive Engagement on Cognitive Decline: Investigating Sex and Life‐Stage Differences

**DOI:** 10.1002/alz70861_108641

**Published:** 2025-12-23

**Authors:** Sarah Goulding, Mahsa Dadar, John AE Anderson, Michael D Oliver, Lisa L. Barnes, Mayra L. Estrella, Cassandra Morrison

**Affiliations:** ^1^ Carleton University, Ottawa, ON Canada; ^2^ Douglas Mental Health University Institute, Montréal, QC Canada; ^3^ Department of Psychiatry, McGill University, Montréal, QC Canada; ^4^ Belmont University, Nashville, TN USA; ^5^ Rush Alzheimer's Disease Center, Rush University Medical Center, Chicago, IL USA; ^6^ Rush Alzheimer’s Disease Center, Rush University Medical Center, Chicago, IL USA

## Abstract

**Background:**

Engaging in cognitively stimulating activity (CSA) enhances cognitive reserve and reduces cognitive decline in aging. However, limited research has compared CSA’s impact across different life stages (e.g., childhood, middle adulthood, older adulthood) or explored whether its protective effects differ by sex. This study was designed to examine (1) sex differences in how CSA engagement across life stages influences cognitive decline, and (2) whether a drop in late‐life engagement impacts cognitive decline compared to consistently high or low engagement throughout life.

**Methods:**

Data from 2,747 participants (2,068 females, 679 males) with 19,523 timepoints and a mean of 6.8 ± 4.5 follow‐up years from the Rush Alzheimer’s Disease Center database were analyzed. CSA engagement levels were categorized as high or low using a median split for early (ages 6 and 12), middle (ages 30–40), and late (ages 65+) life. Participants were grouped by sex and categorized according to their CSA engagement in each life stage and their lifelong engagement patterns: Always Engaged (high engagement in all stages), Always Disengaged (low engagement in all stages), and Disengaged in Late Life (high engagement in early/middle life, low in late life). Linear mixed‐effects models assessed cognitive change over time.

**Results:**

Early‐life engagement did not impact cognitive decline in females or males. In middle‐life, females with high engagement experienced slower decline, while males experienced faster decline than their low engagement counterparts. In late‐life, females and males with high engagement experienced slower decline than their low‐engagement counterparts, with females benefiting more than males (Figure 1). Females who disengaged in late life declined faster than those always disengaged but did not differ from those always engaged. Males who disengaged in late life declined faster than those always engaged but did not differ from those always disengaged (Figure 2).

**Conclusions:**

The relationship between CSA and cognitive decline varies by sex, timing of engagement, and lifelong engagement patterns. These findings emphasize the need to consider these factors in cognitive aging research, and in the development of interventions promoting cognitive health. Although CSA engagement in late‐life is beneficial across sexes, midlife engagement may be particularly important for females.